# Large D-Dimer Fluctuation in Normal Pregnancy: A Longitudinal Cohort Study of 4,117 Samples from 714 Healthy Danish Women

**DOI:** 10.1155/2016/3561675

**Published:** 2016-04-17

**Authors:** Katrine K. Hedengran, Malene R. Andersen, Steen Stender, Pal B. Szecsi

**Affiliations:** Department of Clinical Biochemistry, Copenhagen University Hospital Herlev and Gentofte, 2900 Hellerup, Denmark

## Abstract

*Introduction*. D-dimer levels increase throughout pregnancy, hampering the usefulness of the conventional threshold for dismissing thromboembolism. This study investigates the biological fluctuation of D-dimer in normal pregnancy.* Methods*. A total of 801 healthy women with expected normal pregnancies were recruited. D-dimer was repeatedly measured during pregnancy, at active labor, and on the first and second postpartum days. Percentiles for each gestational week were calculated. Each individual D-dimer was normalized by transformation into percentiles for the relevant gestational age or delivery group. The range in percentage points during the pregnancy and the delivery was calculated, and reference intervals were calculated for each pregnancy trimester, during vaginal delivery and scheduled and emergency cesarean section, and for the first and second day postpartum.* Results*. D-dimer increased during pregnancy; the maximal fluctuation was approximately 20 percentile points in approximately half of the women. In one out of ten women, the D-dimer values fluctuated by more than 50 percentile points.* Conclusions*. Due to the biological variation in D-dimer within each individual woman during normal pregnancy, repeated D-dimer measurements are of no clinical use in the evaluation of thromboembolic events during pregnancy.

## 1. Introduction

Venous thromboembolism (VTE) with or without pulmonary embolism (PE) is a major cause of maternal morbidity and mortality throughout the Western world [1]. VTE occurs in 5–10 per 10,000 pregnancies. Most (approximately 80%) of the pregnancy-associated VTE episodes are isolated deep vein thrombosis (DVT), and 20% of the episodes are associated with PE. Two-thirds of all VTE occur before delivery, with similar frequency during all three trimesters, and the most frequent (80%) location of DVT is in the left leg [2–4].

The diagnosis of VTE in a nonpregnant population is based on a combination of clinical assessments (e.g., Wells score) [5], a D-dimer measurement, and an imaging modality, most frequently ultrasound, or a computed tomography (CT) scan or a MRI scan [6]. Only a minority of pregnant women with suspected VTE actually have the disease; however, the consequences of a misdiagnosis can be serious [2, 3]. Diagnostic strategies in pregnant women with suspected VTE have not been extensively investigated and the role of D-dimer testing remains uncertain. Guidelines for nonpregnant women recommend that VTE can be dismissed with a high degree of certainty when the D-dimer value is below 0.5 mg/L FEU (fibrin equivalent unit), in combination with a low to moderate pretest probability assessment. This threshold provides a high negative predictive value for the detection of VTE, omitting the need for further diagnostic modalities [2, 3].

The conventional D-dimer threshold of 0.5 mg/L is of limited value in pregnant women as D-dimer increases with gestational age. This increase in D-dimer levels is most likely due to continuous coagulation and fibrinolysis during the normal development of the placenta, causing a high frequency of false positive results [6, 7]. Thus, some studies have advocated for a higher D-dimer threshold during pregnancy [8–10], while other studies have suggested the use of a gestational age-specific reference interval [11–13]. Radiation imaging modalities, such as spiral CT and lung ventilation/perfusion scans, are not frequently used during pregnancy due to an assumed increased risk of developmental damage and a lifetime risk of cancer to the fetus [14].

This combination of restricted diagnostic tools presents a challenge during pregnancy, especially due to the increased risk of VTE, which is accentuated by the increasing frequency of obesity and assisted fertilization [15].

Some centers monitor at risk patients during pregnancy with repeated D-dimer measurements and interpret increasing levels as a reason for anticoagulation therapy. In order to be able to evaluate changes in any laboratory value, it is necessary to know the biological variation. According to the Biological Variation Database and the subsequent updates initiated by Ricós et al. the intraindividual variation of D-dimer in a nonpregnant population is about 23% [16]. This value however is not established for pregnant women.

The aim of this study is to investigate the biological variation of D-dimer in normal pregnancy. We hypothesize that D-dimer concentrations fluctuate within a normal pregnancy, rendering the evaluation of changes in D-dimer values useless.

## 2. Materials and Methods

### 2.1. Study Population

**Figure 1:**
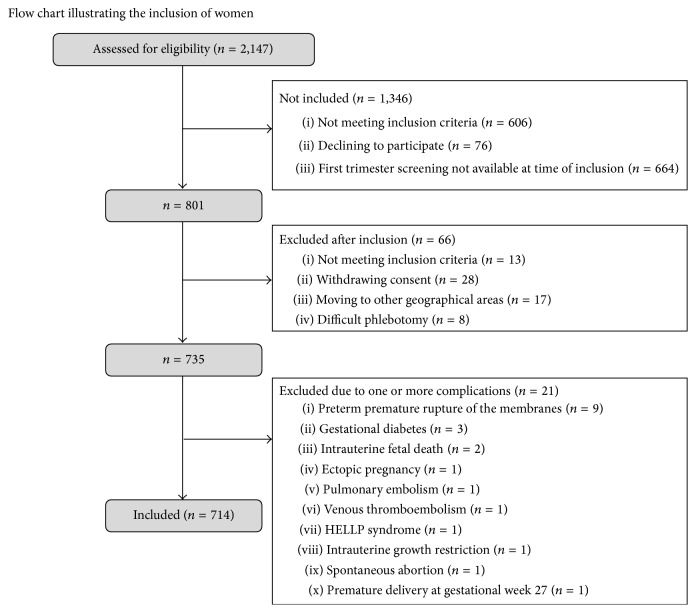
Consort flow chart.

A total of 801 Caucasian women were recruited among 2,147 women attending first trimester screening for Down syndrome from June 2006 to October 2007 at their first contact with Gentofte Hospital. The recruitment process is illustrated in Figure 1 and has been previously described in detail [7, 17, 18]. Exclusion criteria for the 601 women who were not invited to participate were fetal malformation, missed abortion, Down syndrome, other genetic disorders, multiple pregnancies, maternal disease, medicine intake, or previous obstetric complications (preeclampsia, gestational diabetes, preterm delivery, intrauterine growth restriction (IUGR), previous stillbirth, miscarriage after the 12th week of pregnancy, or hypertension). Clinical data were obtained from pregnancy charts and medical records, and the gestational age was estimated using crown-rump length. Each woman was scheduled to provide blood samples seven times during pregnancy and delivery, at gestational weeks 13–20, 21–28, 29–34, and 35–42, during active labor or cesarean, and on the first and second postpartum days. The Scientific Ethics Committee granted approval of this trial (KA 05065) and it was approved by the Danish Data Protection Agency. Oral and written informed consent were obtained from the participants in this study.

### 2.2. Laboratory Testing

Blood samples were collected in tubes containing liquid 0.129 mol/L trisodium citrate (BD Medical Systems, Franklin Lakes, NJ, USA, or Greiner Bio-One, Kremsmünster, Austria), centrifuged at 3,000 ×g for 10 minutes at room temperature, registered, and analyzed on unfrozen material upon arrival at the laboratory as previously described [7] (it was incorrectly reported that D-dimer was analyzed on frozen material). D-dimer was analyzed on the STA-R evolution coagulation analyzer (Diagnostica Stago, Asnieres Sur Seine, France) with STA Liatest D-DI # 00515 traceable to the internal standard and an analytic coefficiency of variation of 2.8% (high level control through one year) according to the manufacturer's specifications and laboratory standards and to the ISO-15189 certification.

### 2.3. Statistical Calculations

The 5th, 25th, 50th, 75th, and 95th percentiles for each gestational week were calculated using the entire cohort before delivery and around partum for vaginal delivery and scheduled and emergency cesarean sections. Each individual D-dimer value was normalized by transformation into percentiles for the relevant gestational age group (or delivery group). The range in percentage points during the pregnancy and delivery was calculated for each individual woman. Reference intervals (2.5th and 97.5th percentiles with 90% confidence intervals (CI)) were calculated for each trimester in pregnancy, for vaginal delivery, for scheduled and emergency cesarean sections, and for the first and second day postpartum using the nonparametric bootstrap method with 500 iterations, performed with RefVal software version 4.11 [9] according to the recommendations of the International Federation of Clinical Chemistry (IFCC) [7]. Outliers were removed with Horn's algorithm (fence factor 1.5), and descriptive 95th interpercentile ranges were used for groups with fewer than 40 measurements. Groups were compared using a one-way analysis of variance (ANOVA). Levene's test for homogeneity of variance and Games-Howell* post hoc* analyses, assuming unequal variances and group size, were used to investigate the nature of any significant differences. A *P* < 0.05 was considered to be statistically significant. Calculations were performed using SPSS 15.2 for Windows (SPSS, Chicago, IL, USA) or Excel 2010 (Microsoft, Redmond, WA, USA).

## 3. Results

A total of 801 pregnant women with a total of 4,310 plasma samples (5.4 samples in average per woman) were enrolled in the study. The women had a mean age of 31.9 years, a mean prepregnant body mass index (BMI) of 22.2 kg/m^2^, and 44% were nulliparous. The mean gestational age at delivery was 283 days, and the mean birth weight was 3,601 grams. All women were expected to have normal pregnancies, and none of the women had known diseases or medicine intake prior to pregnancy. As shown in Figure 1, some 66 women were excluded leaving a total of 714 women (4,117 samples) in the study. Of the 4,117 samples obtained, 2,650 samples were collected during pregnancy, and 1,467 samples were collected during delivery and on the first two days postpartum. Only 657 of the 714 women contributed samples during delivery and postpartum: 534 women (1,199 samples) delivered vaginally, 69 women (150 samples) delivered by scheduled cesarean section, and 54 women (118 samples) delivered by emergency cesarean section.

One 38-year-old woman succumbing to presumed PE was admitted at gestational week 32 in cardiac arrest after jogging and suddenly experiencing shortness of breath. In spite of an extensive resuscitation attempt and emergency cesarean, neither the woman nor the apparently healthy child could be saved. An autopsy was not performed, but the cause of death was presumed to be from a pulmonary embolism. Her D-dimer values were 0.5 and 0.7 mg/L at gestational weeks 15 and 18, respectively.

One 34-year-old woman diagnosed with VTE at gestational week 31 was later diagnosed with protein S deficiency (nonpregnant free protein S of 0.14 IU/mL). She was treated with low molecular weight heparin throughout the rest of the pregnancy, and she experienced an uncomplicated delivery and two more pregnancies and deliveries. Prior to the diagnosis of VTE, her D-dimer values were between 1.0 and 1.3 mg/L.

**Figure 2:**
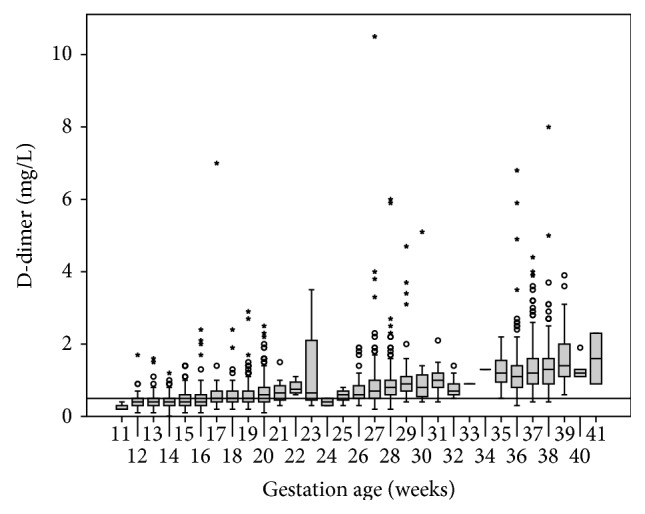
D-dimer during pregnancy. The box plots represent the central distribution of the D-dimer concentration (25th–75th percentile), while the horizontal bar in the middle of each box plot represents the median value. The “whiskers” represent the range of values, excluding outliers (circles) and extreme values (asterisks). The results are provided for each gestational week (11–41). The horizontal line represents the conventional threshold of 0.5 mg/L (FEU).

D-dimer progressively increased throughout the pregnancy (Figure 2). As early as weeks 13–20, more than 25% of the pregnant women had D-dimer levels at or above 0.5 mg/L, and by weeks 36–42, practically all of the pregnant women had values above this conventional threshold (marked for a nonpregnant population with the black line in Figure 2).

**Figure 3:**
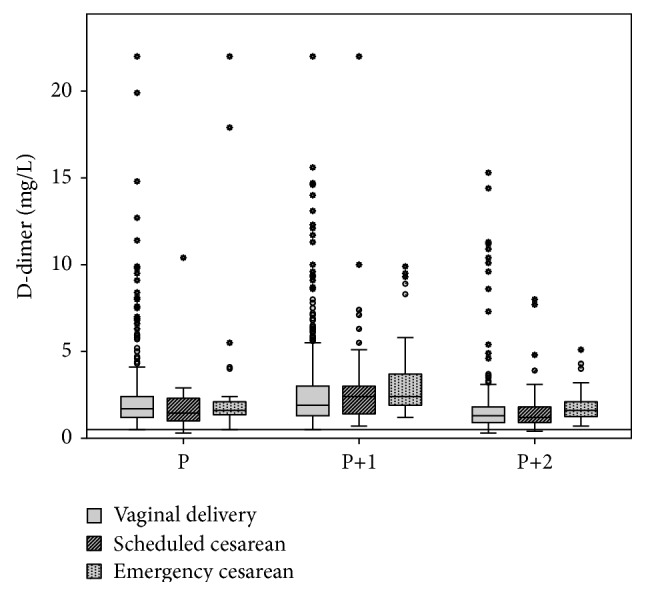
D-dimer during delivery and the early postpartum period. The box plots represent the central distribution of D-dimer concentration (25th–75th percentile), while the horizontal bar in the middle of each box plot represents the median value. The “whiskers” represent the range of values, excluding outliers (circles) and extreme values (asterisks). The results are shown for vaginal delivery, scheduled cesarean section, or emergency cesarean section during delivery (P) and on the first (P+1) and second (P+2) postpartum days. The horizontal line represents the conventional threshold of 0.5 mg/L (FEU).

During delivery and on the first and second days postpartum, D-dimer values were higher than during the gestational period (Figure 3). The D-dimer value (mean; 95% CI) was 2.3 mg/L; 2.1–2.5 at vaginal delivery, 2.8 mg/L; 2.5–3.1 on the first postpartum day, and 1.8 mg/L; 1.6–2.0 on the second postpartum day. For the women delivering by scheduled cesarean, D-dimer value was 1.9 mg/L; 1.3–2.4 during delivery, 3.0 mg/L; 2.2–3.7 on the first day postpartum, and 1.8 mg/L; 1.3–2.2 on the second day postpartum. For the women delivering by emergency cesarean, D-dimer value was 2.7 mg/L; 1.4–3.9 during delivery, 3.3 mg/L; 2.6–4.1 on the first day postpartum, and 1.9 mg/L; 1.6–2.2 on the second day postpartum. There was no significant difference in D-dimer levels among women delivering vaginally or by scheduled or emergency cesarean. A larger proportion of the women that delivered vaginally had outlier or extreme values during and after delivery compared to women who delivered by scheduled or emergency cesarean section, as seen in Figure 3.

**Figure 4:**
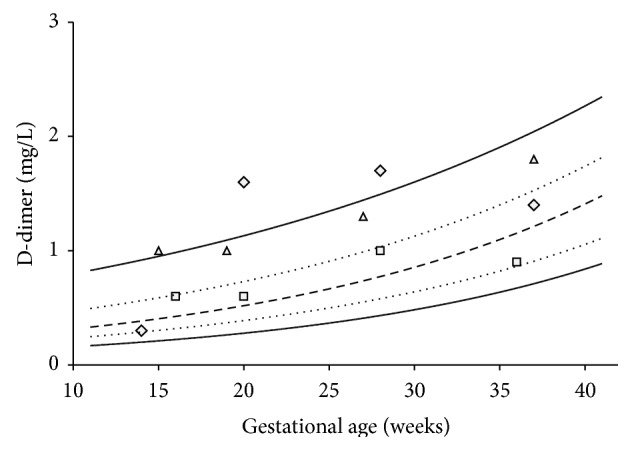
D-dimer concentrations as 5th, 25th, 50th, 75th, and 95th percentiles for each gestational week. D-dimer fluctuations for three randomly selected women are illustrated (square, triangle, and diamond). In spite of all having normal pregnancies, several women had stable high or low D-dimer values, while other had fluctuating D-dimer values throughout the pregnancy. Similar patterns are observed during delivery.

The 5th, 25th, 50th, 75th, and 95th D-dimer percentiles and the exponential trend lines during pregnancy are shown in Figure 4. The individual maximal fluctuation in D-dimer values during pregnancy was approximately 20 percentage points in approximately half of the women, and in one out of ten women, the maximal D-dimer fluctuated by more than 50 percentage points, as seen with the three illustrated examples in Figure 4.

Trimester specific D-dimer reference intervals as 2.5th and 97.5th percentiles with 90% confidence intervals are shown in Table 1. D-dimer reference intervals increase with each trimester, especially the upper reference limit, which was as high as 2.8 mg/L during the third trimester. There was a significant difference in D-dimer levels among trimesters (*P* < 0.001).

Table 2 shows that the D-dimer reference intervals were higher during delivery and the early postpartum period for vaginal delivery than for scheduled and emergency cesareans. The D-dimer levels were notably higher with a peak on the first day postpartum, but there were no statistically significant differences depending on the delivery mode.

## 4. Discussion

Our findings corroborate the opinion that the conventional D-dimer threshold of 0.5 mg/L is of little value in excluding PE or DVT during pregnancy [11–13]. The threshold value has been validated to have a high negative predictive value for VTE in a nonpregnant population with a low to moderate clinical suspicion, rendering compression ultrasounds or ventilation-perfusion CT scans unnecessary [5, 6]. However, as also seen in our population, very few healthy pregnant women have D-dimer values below this threshold. Approximately 25% percent of the women in this study had a D-dimer value above 0.5 mg/L toward the end of the first trimester, and practically no women had D-dimer values below this threshold after gestational week 25.

Some studies have suggested that the threshold during pregnancy should be increased to 1.0 or 2.0 mg/L [8, 9]. A threshold of 1.0 mg/L could offer clinical value until gestational week 30, but approaching full term most healthy women have D-dimer values above this limit. To avoid false positive results requiring further investigations, the threshold should be as high as 2.0 mg/L just prior to delivery. Furthermore, all D-dimer assays are standardized at 0.5 mg/L; however, at higher levels, the concentrations might differ, rendering the determination of a general threshold difficult. Other studies do not recommend the use of D-dimer during pregnancy [14]. In line with previous studies, our data suggests that if a D-dimer threshold during pregnancy is warranted, then it should be gestational age-specific and should be interpreted with great precaution [11–13].

We observe a large individual fluctuation in D-dimer values in healthy women during pregnancy (Figure 4). The individual woman does not necessarily follow a certain gestation age percentile throughout her pregnancy, suggesting that a change in gestation age percentiles is of limited clinical value and cannot be used to predict a thromboembolic event. An individual increase in a D-dimer value should not, by itself, be interpreted as a sign of VTE and should not merit antithrombotic treatment.

Our study focuses on healthy women; thus, negative predictive gestational age reference intervals cannot be established. Only two events of VTE occurred in our study, and neither woman had a D-dimer value that predicted a thromboembolic event. Another limitation of our study is the few samples obtained before gestational week 13. However, our results suggest that extrapolating data from a nonpregnant population to women pregnant in their first trimester is acceptable, but an increased number of false positive results should be expected. This is supported by the recent study of Hammerova et al., who did observe elevated D-dimer values within gestational weeks 10–15 although with a different assay [19].

According to Bayes' theorem, infrequent events lead to a low predictive value [20]. Negative studies including all pregnant women, in which only a small percentage will actually develop VTE, do not rule out that D-dimer values could be of some clinical use in a subgroup with high pretest probability. However, as this event is a rare phenomenon, D-dimer measurement would not provide any clinical help.

The D-dimer value peaks on the first day postpartum, indicating that the coagulation and fibrinolytic systems rapidly return to their normal state, in accordance with previous findings [21]. We do not include measurements later than two days postpartum and thus cannot establish when D-dimer returns to nonpregnant levels. Epiney et al. observed that D-dimer values returned to normal prepregnancy values approximately 6 weeks postpartum [21]. Interestingly, D-dimer rises to equally high values during delivery as those seen after trauma or surgery independent of the delivery method [22].

Several guidelines for diagnosing VTE in pregnant women have been recently published, and the use of D-dimer during pregnancy is not recommended [2, 3, 23, 24]. A D-dimer below 0.5 mg/L in pregnancy may indeed lower the probability of VTE, but there is a high rate of false positives. If the D-dimer value is measured, then the result should be carefully interpreted. Sivandarajah compiled evidence regarding the sensitivity of the D-dimer concentration to rule out VTE in pregnancy and concluded that further research is needed [25].

In women suspected of DVT, ultrasound should be the initial diagnostic test; if the D-dimer test is positive or not measured and the ultrasound does not show signs of DVT but the clinical suspicion is strong, then the ultrasound should be repeated one week later [2]. In women suspected of having PE, a ventilation-perfusion scan is suggested as the initial test. This diagnostic modality is not frequently used during pregnancy due to concerns of side effects for the fetus; however, studies indicate that the risk of fetal damage and maternal exposure are negligible and outweighed by the benefits of a correct diagnosis of PE [3, 24, 26].

## 5. Conclusion

Our results confirm that the conventional negative predictive threshold of 0.5 mg/L for D-dimer is of limited use during pregnancy due to a high number of false positives. An elevated D-dimer threshold throughout the pregnancy cannot be recommended. A gestational age-specific reference interval is required, and even then, D-dimer values should be interpreted with precaution due to a large number of outliers. Our results also suggest that D-dimer levels fluctuate in the individual woman throughout the pregnancy in a high proportion of women, rendering repeated D-dimer measurements of no clinical use in excluding VTE.

## Figures and Tables

**Table 1:** Gestational age-specific D-dimer reference intervals.

	2.5th percentile (90% CI)	97.5th percentile (90% CI)	Samples (*n*)	Outliers (*n*)
1st trimester Gestational weeks <15	**0.2** (0.2-0.2)	**0.9** (0.8–0.9)	222	13
2nd trimester Gestational weeks 15–27	**0.2** (0.2-0.2)	**1.5** (1.4–1.6)	1412	12
3rd trimester Gestational weeks >27	**0.4** (0.4–0.5)	**2.8** (2.6–3.1)	971	20

The 2.5th and 97.5th percentiles (bold) with 90% confidence intervals (in brackets) are given. Outliers were removed using Horn's algorithm with a fence factor of 1.5.

**Table 2:** The 95% D-dimer reference intervals during delivery and postpartum according to the mode of delivery.

	2.5th percentile (90% CI)	97.5th percentile (90% CI)	Samples (*n*)	Outliers (*n*)
Vaginal delivery				
At delivery	**0.7** (0.7–0.8)	**7.5** (6.3–8.1)	443	10
1st day postpartum	**0.8** (0.7–0.8)	**11.7** (9.3–14.0)	438	1
2nd day postpartum	**0.6** (0.6-0.6)	**5.1** (4.3–9.6)	300	7
Scheduled cesarean				
At delivery	0.6^*∗*^	3.3^*∗*^	36	2
1st day postpartum	**0.9** (0.7–1.1)	**8.5** (6.3–10)	60	1
2nd day postpartum	**0.6** (0.6–0.8)	**7.0** (3.9–7.7)	49	2
Emergency cesarean				
At delivery	**0.8** (0.8–1.0)	**5.5** (4.0–5.5)	40	3
1st day postpartum	**1.2** (1.2–1.5)	**9.9** (9.3–9.9)	40	0
2nd day postpartum	1.3^*∗*^	9.5^*∗*^	35	0

D-dimer reference intervals in mg/L (FEU) for delivery and early postpartum period given as 2.5th and 97.5th percentiles (bold) with 90% confidence intervals (in brackets). ^*∗*^For groups containing fewer than 40 samples, descriptive 5th and 95th percentiles are provided without any confidence intervals. Outliers were removed using Horn's algorithm with a fence factor of 1.5.

## References

[1] Chang J., Elam-Evans L. D., Berg C. J. (2003). Pregnancy-related mortality surveillance—United States, 1991–1999. *Morbidity and Mortality Weekly Report*.

[2] Bates S. M., Jaeschke R., Stevens S. M. (2012). Diagnosis of DVT: antithrombotic therapy and prevention of thrombosis, 9th ed: American college of chest physicians evidence-based clinical practice guidelines. *Chest*.

[3] Bourjeily G., Paidas M., Khalil H., Rosene-Montella K., Rodger M. (2010). Pulmonary embolism in pregnancy. *The Lancet*.

[4] James A. H. (2012). Prevention and treatment of venous thromboembolism in pregnancy. *Clinical Obstetrics and Gynecology*.

[5] Wells P. S., Anderson D. R., Rodger M. (2003). Evaluation of D-dimer in the diagnosis of suspected deep-vein thrombosis. *The New England Journal of Medicine*.

[6] Righini M., Perrier A., De Moerloose P., Bounameaux H. (2008). D-Dimer for venous thromboembolism diagnosis: 20 years later. *Journal of Thrombosis and Haemostasis*.

[7] Szecsi P. B., Jørgensen M., Klajnbard A., Andersen M. R., Colov N. P., Stender S. (2010). Haemostatic reference intervals in pregnancy. *Thrombosis and Haemostasis*.

[8] Kawaguchi S., Yamada T., Takeda M. (2013). Changes in d-dimer levels in pregnant women according to gestational week. *Pregnancy Hypertension*.

[9] Kovac M., Mikovic Z., Rakicevic L. (2010). The use of D-dimer with new cutoff can be useful in diagnosis of venous thromboembolism in pregnancy. *European Journal of Obstetrics Gynecology and Reproductive Biology*.

[10] Kline J. A., Williams G. W., Hernandez-Nino J. (2005). D-dimer concentrations in normal pregnancy: new diagnostic thresholds are needed. *Clinical Chemistry*.

[11] Ercan Ş., Özkan S., Yücel N., Orçun A. (2015). Establishing reference intervals for D-dimer to trimesters. *Journal of Maternal-Fetal and Neonatal Medicine*.

[12] Murphy N., Broadhurst D. I., Khashan A. S., Gilligan O., Kenny L. C., O'Donoghue K. (2015). Gestation-specific D-dimer reference ranges: a cross-sectional study. *BJOG*.

[13] Wang M., Lu S., Li S., Shen F. (2013). Reference intervals of D-dimer during the pregnancy and puerperium period on the STA-R evolution coagulation analyzer. *Clinica Chimica Acta*.

[14] McLintock C., Brighton T., Chunilal S. (2012). Recommendations for the diagnosis and treatment of deep venous thrombosis and pulmonary embolism in pregnancy and the postpartum period. *Australian and New Zealand Journal of Obstetrics and Gynaecology*.

[15] Henriksson P., Westerlund E., Wallén H., Brandt L., Hovatta O., Ekbom A. (2013). Incidence of pulmonary and venous thromboembolism in pregnancies after in vitro fertilisation: cross sectional study. *British Medical Journal*.

[16] Ricós C., Alvarez V., Cava F. (1999). Current databases on biological variation: pros, cons and progress. *Scandinavian Journal of Clinical and Laboratory Investigation*.

[17] Klajnbard A., Szecsi P. B., Colov N. P. (2010). Laboratory reference intervals during pregnancy, delivery and the early postpartum period. *Clinical Chemistry and Laboratory Medicine*.

[18] Szecsi P. B., Andersen M. R., Bjørngaard B., Hedengran K. K., Stender S. (2014). Cancer antigen 125 after delivery in women with a normal pregnancy: a prospective cohort study. *Acta Obstetricia et Gynecologica Scandinavica*.

[19] Hammerova L., Chabada J., Drobny J., Batorova A. (2014). Longitudinal evaluation of markers of hemostasis in pregnancy. *Bratislavské Lekárske Listy*.

[20] Medow M. A., Lucey C. R. (2011). A qualitative approach to Bayes' theorem. *Evidence-Based Medicine*.

[21] Epiney M., Boehlen F., Boulvain M. (2005). D-dimer levels during delivery and the postpartum. *Journal of Thrombosis and Haemostasis*.

[22] Johna S., Cemaj S., O'Callaghan T., Catalano R. (2002). Effect of tissue injury on D-Dimer levels: a prospective study in trauma patients. *Medical Science Monitor*.

[23] Leung A. N., Bull T. M., Jaeschke R. (2011). An official American Thoracic Society/Society of Thoracic Radiology clinical practice guideline: evaluation of suspected pulmonary embolism in pregnancy. *American Journal of Respiratory and Critical Care Medicine*.

[24] Conti E., Zezza L., Ralli E. (2014). Pulmonary embolism in pregnancy. *Journal of Thrombosis and Thrombolysis*.

[25] Sivandarajah S. (2011). Towards evidence-based emergency medicine: best BETs from the Manchester Royal Infirmary. BET 4: current evidence does not support the use of a negative D-dimer to rule out suspected pulmonary embolism in pregnancy. *Emergency Medicine Journal*.

[26] Middeldorp S. (2013). Thrombosis in women: what are the knowledge gaps in 2013?. *Journal of Thrombosis and Haemostasis*.

